# Disturbing miR-182 and -381 Inhibits BRD7 Transcription and Glioma Growth by Directly Targeting *LRRC4*

**DOI:** 10.1371/journal.pone.0084146

**Published:** 2014-01-03

**Authors:** Hailin Tang, Zeyou Wang, Qing Liu, Xiaoping Liu, Minghua Wu, Guiyuan Li

**Affiliations:** 1 Cancer Research Institute, Central South University,Key Laboratory of Carcinogenesis and Cancer Invasion, Ministry of Education, Key Laboratory of Carcinogenesis, Ministry of Health, Changsha, China; 2 Sun Yat-Sen University Cancer Center, State Key Laboratory of Oncology in South China, Collaborative Innovation Center for Cancer Medicine, Guangzhou, China; 3 The Center for Skull Base Surgery and Neurooncology, Hunan Province, Changsha, China; H.Lee Moffitt Cancer Center & Research Institute, United States of America

## Abstract

Inactivated *LRRC4* has been clinically detected in gliomas, and promoter hypermethylation has been implicated as the mechanism of inactivation in some of those tumors. Our previous researches indicated that *LRRC4* is a target gene of miR-381, the interaction of miR-381 and *LRRC4* is involved in glioma growth. In this study, we demonstrate that *LRRC4* is a target gene of the other microRNA, miR-182. We found that the high expression of miR-182 and miR-381 in gliomas are involved in pathological malignant progression. The silencing of miR-182 and miR-381 inhibited the proliferation *in vitro* and growth of glioma cell with *in vivo* magnetic resonance imaging by intracranial transplanted tumor model in rats. We also demonstrated that BRD7, a transcriptional cofactor for p53, is highly expressed and negatively correlated with *LRRC4* expression in gliomas. Disturbing miR-182 and miR-381 affected transcriptional regulation of the *BRD7* gene. This finding was verified by ectopic overexpression of *LRRC4* or restoration of endogenous *LRRC4* expression by treatment with the DNA demethylating agent 5-Aza-dC. Taken together, miR-182 and miR-381 may be a useful therapeutic target for treatment of glioma.

## Introduction

Gliomas are the most common type of primary brain malignancies and are characterized by well-defined genetic alterations and frequent chromosomal aberrations that are associated with tumorigenesis[1].The chromosome region 7q31-32 is thought to contain multiple tumor-suppressor genes involved in the pathogenesis of head and neck tumors, one of which is the leucine-rich repeat C4 (LRRC4) gene [2]. Our previous studies indicated that *LRRC4* gene expression was highly specific to brain tissue[3], and that the gene product behaved as a tumor suppressor in the pathogenesis of malignant gliomas [4]. Inactivated *LRRC4* has been clinically detected in gliomas, and promoter hypermethylation has been implicated as the mechanism of inactivation in some of those tumors[5]. When *LRRC4* expression was induced in the human U251 glioma cell line, the Ras/p-c-Raf/ERK/MAPK and PI-3K/AKT signaling pathways were found to be down-regulated, which in turn inhibited cell proliferation and invasion [6], [7][.Functional studies of mouse *LRRC4* (*mLRRC4*) determined that the gene was crucial for fetal brain development, as it mediated cell cycle progression and neuron and glia cell differentiation [2], [8]. Therefore, *LRRC4* is considered an important regulator of cell maintenance, growth, and differentiation in the nervous system. However, the molecular mechanism by which *LRRC4* regulates glioma tumorigenesis has not been fully elucidated.

An increasing number of studies have demonstrated that tumour suppressive miRNAs are frequently downregulated in tumours, whereas oncogenic miRNAs are frequently upregulated[9]. We previously reported that miR-200b is greatly downregulated in gastric cells and tissues and inhibits gastric cancer proliferation and invasion[9], [10]. In contrast, the oncogenic miR-183/182/96 cluster of miRNAs is upregulated in a variety of tumours[9], [11], [12], and we previously reported that miR-183/182/96 cluster regulates oxidative apoptosis and sensitises gliomal cells to chemotherapy[13]. miRNA-mediated gene regulation has been demonstrated to contribute to numerous life-sustaining biological processes, including cell proliferation, differentiation, development, and metabolism, and to pathogenic processes, such as cancer formation[14]–[16].Computational algorithms have been developed to identify potential gene targets of miRNAs, either by using the miRNA to find genes that harbor the predicted target sequence or by using a known gene to search for a miRNA that would match its nucleotide sequence. When the glioma-related *LRRC4* gene was queried by TargetScan and PicTar software, it was identified as a target gene of miR-182 and miR-381. We have confirmed that *LRRC4* is a target gene of miR-381, at the same time, overexpression of *LRRC4* also downregulated the expression of miR-381 in glioma cells, the interaction of miR-381 and *LRRC4* is involved in glioma growth[17]; when the miRNAs were used for query, it was found that miR-381 targets both *LRRC4* and *BRD7*, which is cloned by our laboratory [18].

In our laboratory, *BRD7* has been reported as absent or expressed at unusually low levels in nasopharyngeal carcinoma (NPC) biopsies, and overexpression of *BRD7* inhibits NPC cell growth and arrest cells in the G0/G1 phase of the cell cycle[18]. Within the *BRD7* promoter, a Sp1/Myc-Max overlapping site has been defined as a key regulatory element; Sp1 binding leads to slightly positive regulation of promoter activity, while c-Myc binding negatively regulates activity[19]. Moreover, in other laboratories, BRD7 was found to act as a transcriptional cofactor for p53[20], and BRD7 is a candidate tumour suppressor gene required for p53 function [21]. BRD7 interacts with PRMT5 and PRC2, and is involved in transcriptional repression of their target genes [22]. But, the expression and function of *BRD7* in gliomas have yet to be reported.

On the basis of our previous research that *LRRC4* is a target gene of miR-381, we confirmed *LRRC4* is also the target gene of miR-182. BRD7 isn’t the target gene of miR-381, but miR-381 did downregulated the expression of BRD7. We further found that the expression of miR-182 and miR-381 or *BRD7* and *LRRC4* were negatively correlated with the pathological progression of gliomas. Both ectopic *LRRC4* overexpression, or the restoration of LRRC4 endogenous expression inhibited the expression of *BRD7*, locked-nucleic-acid (LNA)-mediated miR-182 and -381 silencing specifically targeted the *LRRC4* and led to perturbations in the Ras/Raf/ERK/MAPK and PI-3K/AKT signaling pathways and to down-regulation of BRD7, which inhibited glioma growth. The study not only demonstrated the mechanism of tumor suppressor gene *LRRC4* involved in the glioma progression, but also provided the therapy targets for glioma.

## Results

### LRRC4 is a bona fide target of miR-182

**Figure 1:**
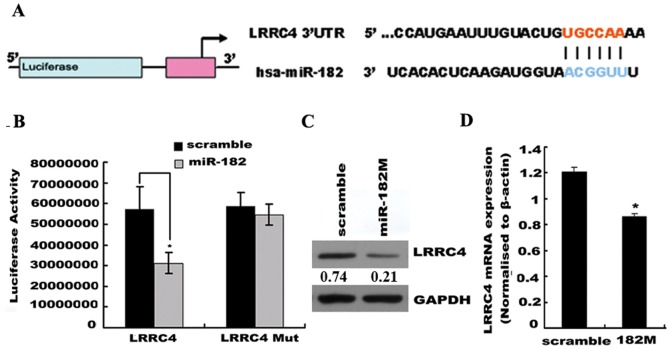
*LRRC4* is a target gene of miR-182. (A) Schema of the interaction sites between miR-182 and the 3′-UTRs of *LRRC4* (B) (B) Luciferase assay of U251 glioma cells co-transfected with pMIR-REPORT–WT/mutant 3′-UTR *LRRC4* and miR-182 or scrambled control as indicated. * *p*<0.05. (C) Western blot showing the protein expression of *LRRC4* after miR-182 was transfected into U251/L cells for 48 h. miR-182 mimics inhibited the protein expression of *LRRC4*. GAPDH was used as a loading control. (D) qRT-PCR showing the mRNA level of *LRRC4* after miR-182 mimic was transfected into U251/L cells for 48 h. miR-182 mimic down-regulated the mRNA level of *LRRC4*. * *p*<0.05.

The miRNA target prediction programs TargetScan and PicTar identified miR-182 interaction sites in the 3′-UTR of *LRRC4* (Figure 1A). We confirmed that *LRRC4* is a common bona fide target of miR-182 (Figure 1B) by performing luciferase reporter assays. *LRRC4* is specifically expressed in normal brain tissues and is down-regulated or deleted in primary brain tumor biopsies (up to 87.5% in gliomas) and glioma cell lines [4]. Because cell lines expressing high levels of LRRC4 are difficult to obtain, we chose to use U251 cells stably transfected with *LRRC4* to generate a model of *LRRC4* highly expressing cells (U251/L cells) [4]. Transfection of the miR-182 mimics into U251/L cells resulted in a marked reduction of *LRRC4* at both the protein (Figure 1C) and mRNA levels, as compared with the control (scrambled) transfection (Figure 1D). Although *BRD7* was predicted as a putative target of miR-381, the miRNA did not combine with the 3′-UTR of *BRD7* and the gene sequence was not a *bona fide* target. However, overexpression of miR-381 mimics did downregulated the expression of BRD7 (Figure S1).

### miR-182, miR-381, BRD7 are inversely correlated with LRRC4 expression in gliomas

**Figure 2:**
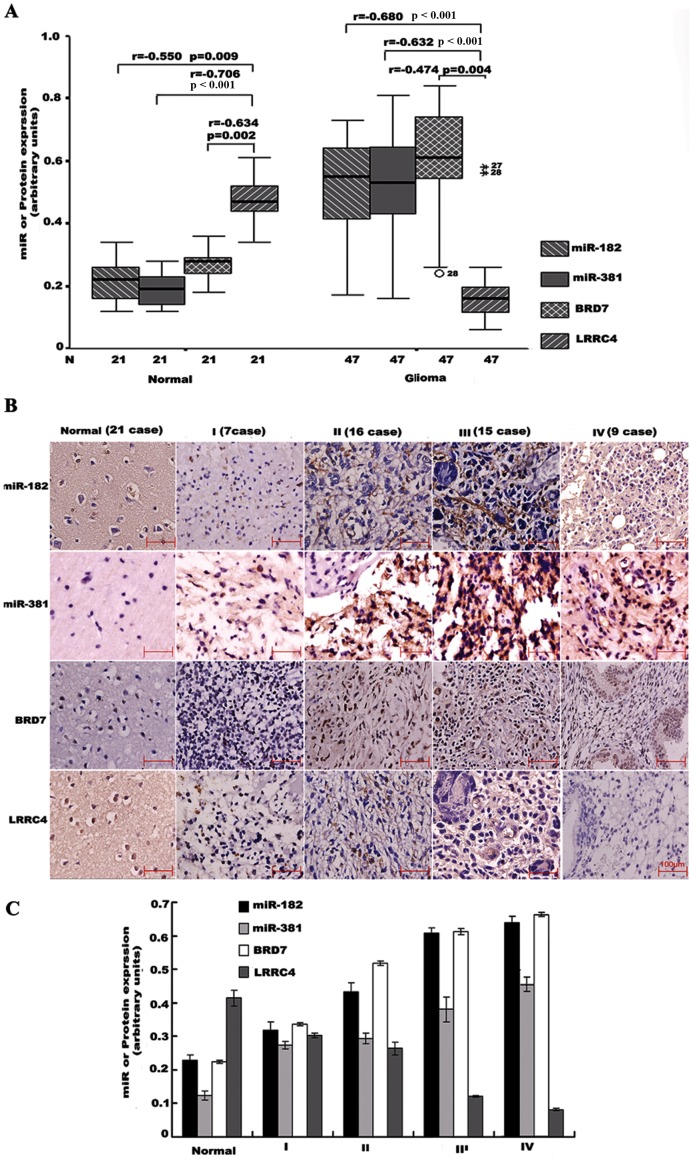
miRNA-182 and miR-381 or *BRD7* expression is inversely related to *LRRC4* expression in gliomas. (A) miRNA-182 and miR-381 or *BRD7* expression is inversely related to *LRRC4* expression in glioma tissues. miR-182 and miR-381 expression levels were assessed by ISH. IHC was used to detect the protein expression of *LRRC4* and *BRD7*. (B) miRNA-182 and miR-381 or *BRD7* expression is inversely related to *LRRC4* expression in normal brain and WHO grade I, II, III astrocytomas, and grade IV glioblastoma. (C) The total gray value of (B). Image analysis and total gray value were estimated by the GSM-2000P pathology image analysis system.

To evaluate the relevance of the endogenous expressions of miR-182, miR-381, *BRD7*, and *LRRC4*, we assessed their expressions in human glioma tissues, as well as in normal brain tissues. ISH analysis of miR-182 and miR-381 and IHC analysis of BRD7 and LRRC4 showed that all 47 primary gliomas had elevated levels of miR-182, miR-381, and BRD7 (except for one case), and decreased levels of LRRC4 (except for two cases), as compared to levels detected in the 21 normal brain tissues. We found that the expressions of miR-182, miR-381 or BRD7 proteins were inversely correlated with expression of LRRC4 in glioma tissues and normal brain tissues (Figure 2A). More importantly, we also found that miR-182, miR-381 and BRD7 were inversely correlated with LRRC4 in astrocytomas of various pathological grades (Figure 2B, C), and the extent of correlation increased from WHO grade I to IV. In contrast, the expression of *LRRC4* in astrocytomas gradually decreased with increase in WHO grade, from I to III, and was completely absent in grade IV astrocytomas (Figure 2B, C). In addition, there was a positive correlation found between expressions of miR-182 or miR-381 and *BRD7* in all glioma tissues, and normal brain tissues. As shown in Figure S2, qRT-PCR was used to furhter verify the expression levels of miR-182 and miR-381 in 19 normal brain tissue samples and 67 primary gliomas. The results indicated that expression change of miR-182 and miR-381 in normal brain tissue and different grade gliomas (I: 10 cases; II: 22 cases; III: 23 cases; IV: 12 cases) was consistent to that detected by ISH (Figure 2 A-C).

### miR-182 and miR-381 silencing inhibited glioma tumorigenicity and induced differentiation

**Figure 3:**
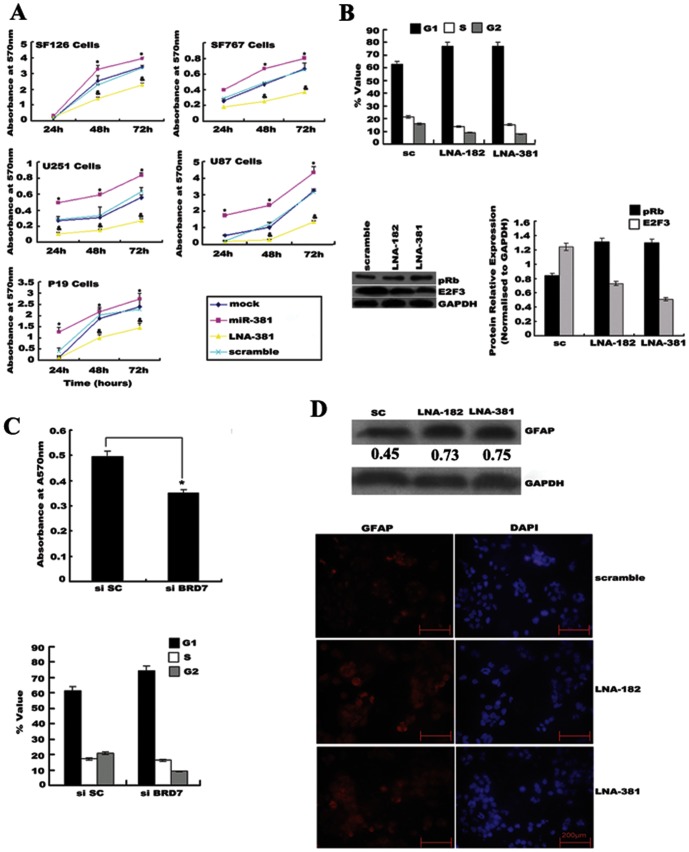
LNA-anti-miR-182 and -381 had anticancer effects on glioma cells and subcutaneously transplanted tumors in nude mice. (A) MTT assays confirmed the effects of the ectopic miR-381 mimic or LNA-mediated miR-381 silencing on glioma cell proliferation. The ectopic miR-381 mimic promoted the proliferation of glioma cells and LNA-mediated miR-381 silencing inhibited it. * *p*<0.05 compared with control (mock or scrambled). (B) LNA-mediated miR-182 and -381 silencing blocked cell cycle progression in the G0/G1 phase, induced pRb expression, and decreased E2F3 expression. (C) *BRD7* silencing inhibited the proliferation of glioma cells and blocked the cell cycle in the G0/G1 phase. * *p*<0.05 compared with control (siRNA scrambled). (D) LNA-mediated miR-182 and -381 silencing up-regulated GFAP expression in U251 cells (top, Western blot; bottom, indirect immunofluorescence). Expression of GAPDH was used as an internal loading control for Western blotting. DAPI staining was used as an internal control for immunofluorescence.

To directly test the functional roles of miR-182 and miR-381 in cell proliferation, ectopic miR-182 and miR-381 mimics or LNA-anti-miR-182 and -381 oligonucleotides were transfected into multiple glioma cell lines. There were obvious increases in the proliferation of U251, U87, and P19 cells by 24 h after transfection with the miR-182 and -381 mimics. and an increase in the proliferation of SF126 and SF767 cells by 48 h, as compared with the control (mock-transfected) cells and cells transfected with a scrambled sequence (*t-*test, *p*<0.01; Figure 3A). In contrast, LNA-anti-miR-381 and miR-381 mimics transfection significantly decreased the proliferation of all of these cell lines within 48 h (Figure 3A). Similar patterns were observed for ectopic miR-182 expression or LNA-mediated miR-182 silencing (data not shown). A cell-cycle analysis showed that transfection of LNA-anti-miR-182 and -381 into U251 cells led to a decrease in the proportion of cells in the S and G2-M phases and a corresponding increase in cells in the G1 phase. The LNA-anti-miR-182 and -381 transfections also increased Rb expression and decreased E2F3 expression (Figure 3B). Ectopic *LRRC4* expression [2]–[4], 5-Aza-dC treatment [5], and *BRD7*-siRNA transfection each inhibited cell proliferation and induced cell cycle arrest at G0/G1 in glioma cells (Figure 3C). However, compared with treatment with either LNA-anti-miR-182, LNA-anti-miR-381 or 5-Aza-dC alone, the combination treatment of LNA-anti-miR-182/5AZa, LNA-anti-miR-381/5AZa, or LNA-anti-miR-182/LNA-anti-miR-381 did not induce any obvious differences in the growth inhibition of U251 cells (Figure S3).

Encouraged by these *in vitro* results, we investigated the feasibility of using the LNA-anti-miR-182 and -381 oligonucleotides as an *in vivo* anti-tumor agent. U251 cells were subcutaneously injected into nude mice to yield tumors that were then treated by direct intratumoral injection as soon as they became palpable. The growth curves of treated *vs*. control-treated tumors are compared in Supplementary Figure S1B. As shown, the growth curves slowly diverged, reaching statistical significance at 21 days after the first antagomir injection (*t*-test, *p*<0.01).

Treatment of U251 cells with LNA-anti-miR-182 or -381 led to differentiation into astrocyte-like cells, as demonstrated by the induced expression of glial fibrillary acidic protein (GFAP). In contrast, transfection of the LNA-scrambled control resulted in a low level of GFAP expression. There were no differences in the levels of detected GAPDH or 4′, 6′-diamidino-2-phenylindole (DAPI) nuclear counterstaining between the different groups (Figure 3D).

**Figure 4:**
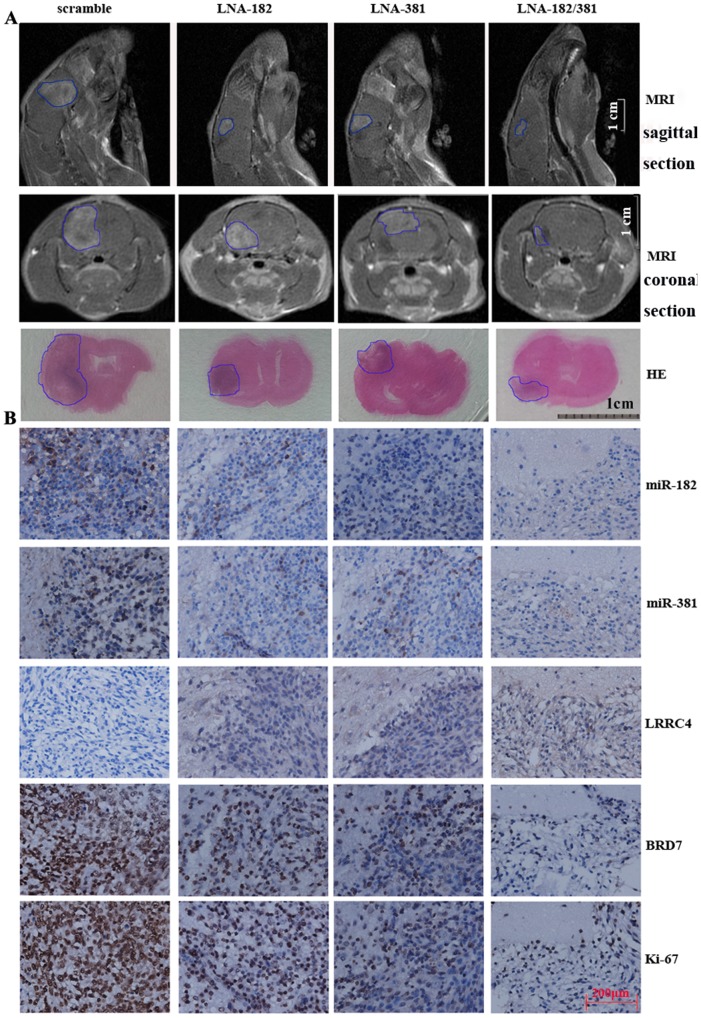
LNA-anti-miR-182 and -381 had anticancer effects on intracranial transplanted tumors by surpassing the blood-brain barrier. (A) Intraperitoneal injection of LNA-anti-miR-182 and/or -381 oligonucleotides inhibited the growth of intracranial transplanted tumors in Sprague-Dawley rats (top and middle, MRI; bottom, HE staining of coronal section). (B) LNA-anti-miR-182 and/or -381 oligonucleotides reduced expression of miR-182 and -381 (ISH), increased expression of LRRC4, and reduced expression of BRD7 and Ki-67 (IHC).

Next, we investigated whether the LNA-anti-miR-182 and/or -381 was able to surpass the blood-brain barrier and inhibit growth of the intracranial transplanted tumors. C6 glioma cells tumors were intracranially transplanted into the brain parenchyma of Sprague-Dawley rats. The transplanted rats were treated with intraperitoneal injections of 200 µL of 0.9% saline solution containing: 2 µg of scrambled, LNA-anti-miR-182, or LNA-anti-miR-381, or 1 µg of LNA-anti-miR-182+1 µg of LNA-anti-miR-381 over one day. Magnetic resonance imaging (MRI) revealed that administration of miR-182 or miR-381 inhibitors was accompanied by significantly reduced growth of the intracranial transplanted tumors. The inhibitory effect of the combination treatment with miR-182+miR-381 inhibitors was more robust than single treatments (Figures 4A and S4A). ISH and IHC indicated that LNA-anti-miR-381 and/or LNA-anti-miR-182 treatment increased the expression of LRRC4 and reduced the expression of BRD7 (Figures 4B and S4B).

### miR-182 and miR-381 silencing induced BRD7 down-regulation by targeting LRRC4

**Figure 5:**
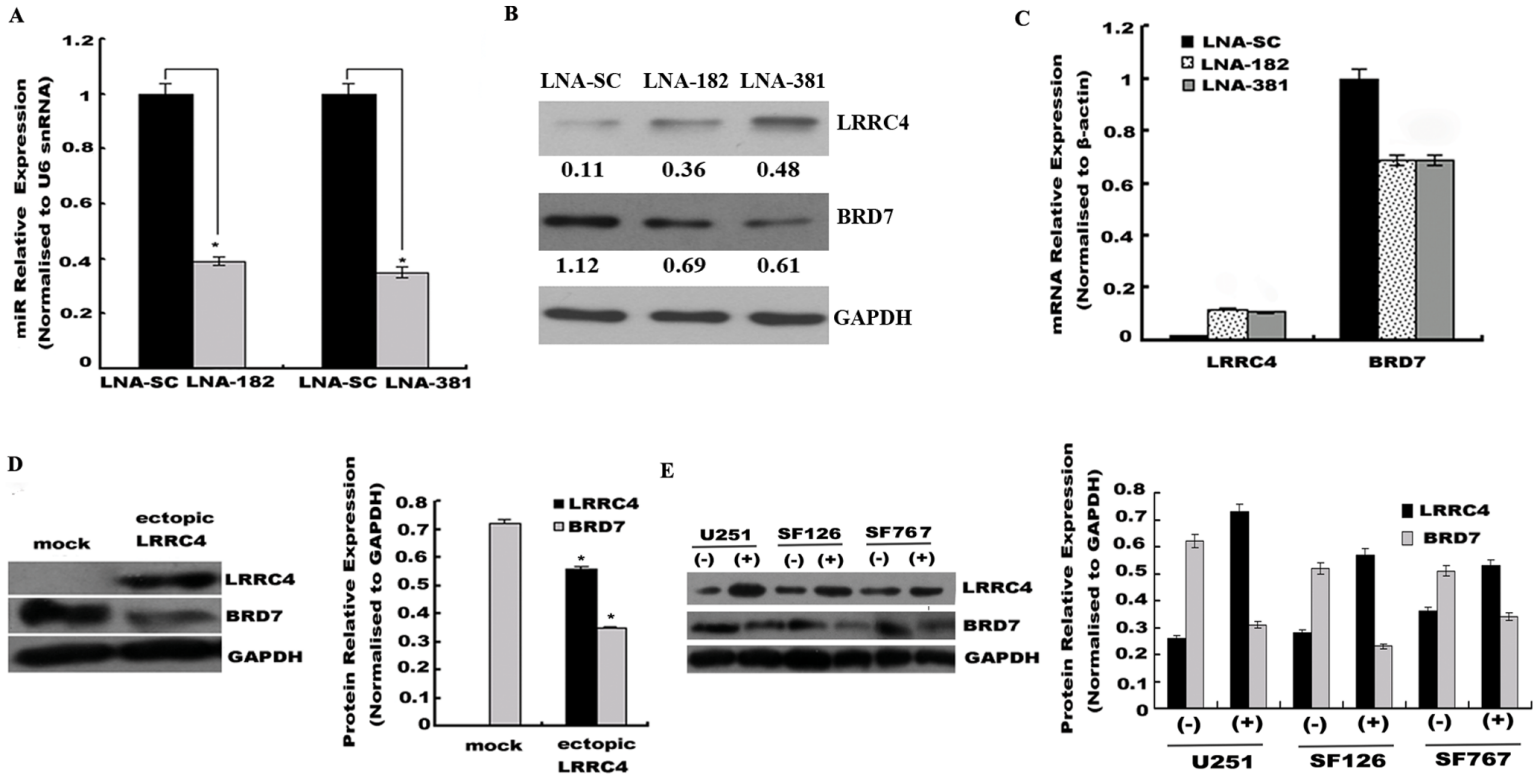
LNA-anti-miR-182 and -381 induces *LRRC4* up-regulation and *BRD7* down-regulation. (A) qRT-PCR showing down-regulation of miR-182 and miR-381 in U251 cells after LNA-anti-miRs transfection. * *p*<0.05. (B) LNA-mediated miR-182 and -381 silencing restored endogenous levels of *LRRC4* protein and decreased BRD7 expression. U251 cells were transfected with either LNA-scrambled, LNA-anti-miR-182 or -381 for 48 h. *LRRC4* and *BRD7* expression was assessed by Western blot. GAPDH was used as a loading control. (C) qRT-PCR confirmed re-expression of *LRRC4* and decreased *BRD7* expression after LNA-anti-miR-182 and -381 transfection. (D) Ectopic *LRRC4* expression decreased endogenous levels of *BRD7* protein in U251 cells. *LRRC4* and *BRD7* expression were assessed by Western blot (left) and gray image scanning (right). * *p*<0.05 compared with mock (control). (E) 5-Aza-dC restored endogenous levels of *LRRC4* protein and decreased that of *BRD7* expression in U251, SF126, and SF767 cells. *LRRC4* and *BRD7* expressions were assessed by Western blot (left) and gray image scanning (right). * *p*<0.05 compared with LNA-scrambled control.

To further test the effects of miR-182 and miR-381 on endogenous *LRRC4* and *BRD7* expressions, we used simple systemic delivery of an unconjugated, PBS-formulated LNA-anti-miR to antagonize expression of endogenous miR-182 and -381 in U251 cells (Figure 5A). Western blotting indicated that LNA-mediated silencing of miR-182 and -381 led to recovery of *LRRC4* expression and reduced *BRD7* expression (Figure 5B). These results were confirmed by qRT-PCR (Figure 5C).

Previous studies had indicated that *LRRC4* was down-regulated in glioma biopsies and cell lines [2], [4], and that methylation of the *LRRC4* promoter was one of the reasons for *LRRC4* inactivation in some gliomas [5]. To test if the observed down-regulated of *BRD7* was *LRRC4*-dependent, we analyzed the effects of ectopic *LRRC4* expression and 5-Aza-dC demethylation treatment on *BRD7* expression. Ectopic *LRRC4* overexpression in U251 cells (Figure 5D) and 5-Aza-dC-mediated re-expression of *LRRC4* in U251, SF126, and SF767 cells (Figure 5E) led to suppression of *BRD7* expression, suggesting that miR-182 and miR-381 silencing inhibited *BRD7* expression by targeting *LRRC4*.

### miR-182 and miR-381 silencing regulated AP2/SP1/E2F6/c-Myc-mediated BRD7 transcription induced by LRRC4-mediated K-Ras/c-Raf/ERK/MAPK and PI-3K/AKT signaling pathways

**Figure 6:**
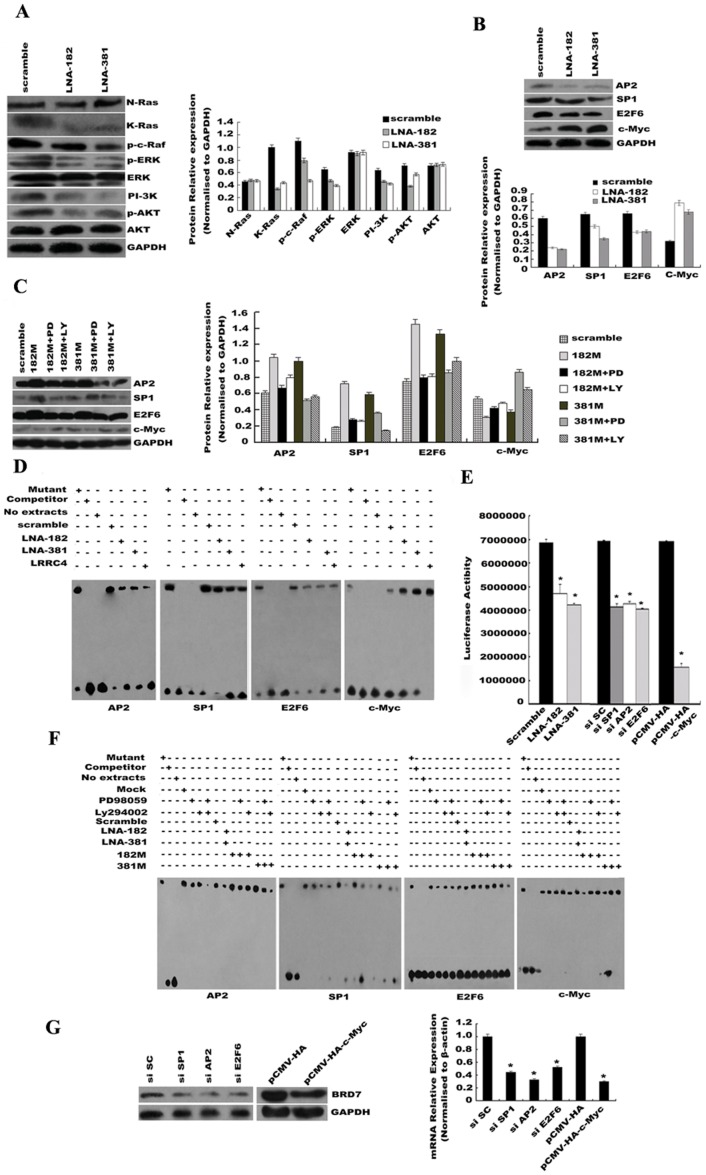
LNA-anti-miR-182 and -381 suppressed the promoter activity of *BRD7* by down-regulating AP2, SP1, and E2F6, and up-regulating c-Myc. (A) LNA-mediated miR-182 and -381 silencing down-regulated expression of K-Ras, p-c-Raf, pERK, PI-3K, and pAKT, but the silencing had no effect on N-Ras, total ERK and AKT expression, as shown by Western blot (left) and gray image scanning (right). (B) LNA-mediated miR-182 and -381 silencing down-regulated the expression of AP2, SP1, and E2F6, and up-regulated the expression of c-Myc, as shown by Western blot (top) and gray image scanning (bottom). (C) PD98059 or LY294002 reversed the miR-182 and miR-381 mimics-induced expression of AP2, SP1, E2F6, and c-Myc. AP2, SP1, E2F6, and c-Myc expression were assessed by Western blot (left) and gray image scanning (right). (D) EMSA confirmed that LNA-mediated miR-182 and -381 silencing or ectopic *LRRC4* expression promoted the *BRD7* promoter association of c-Myc and disrupted that of AP2, SP1, and E2F6. Mutant, nuclear protein +200×mutant probe + wild biotin-probe; Competitor, nuclear protein +200×competitor cold probe + wild biotin-probe; No extracts, no nuclear protein + wild biotin-probe; Scrambled, nuclear protein of transfected miRNA negative control + wild biotin-probe; LNA-182, nuclear protein of transfected LNA-miR-182 inhibitors + wild biotin-probe; LNA-381, nuclear protein of transfected LNA-miR-381 inhibitors + wild biotin-probe; LRRC4, nuclear protein of transfected LRRC4 gene + wild biotin-probe. (E) Luciferase assays confirmed the inhibition of LNA-mediated miR-182 and -381 silencing, or siRNA of AP2, SP1, and E2F6, and c-Myc overexpression on the promoter activity of *BRD7* gene. * *p*<0.05 compared with the control (siRNA including SP1, AP2, and E2F6 *vs.* siRNA scrambled or pCMV-HA-c-Myc *vs.* pCMV-HA). (F) EMSA indicated that PD98059 and LY294002 reversed the association of AP2, SP1, and E2F6 or c-Myc with the *BRD7* promoter that was induced by miR-182 and miR-381. Mutant, nuclear protein+200×mutant probe + wild biotin-probe; Competitor, nuclear protein +200×competitor wild probe + wild biotin-probe; No extracts, no nuclear protein + wild biotin-probe; Mock, nuclear protein+ wild biotin-probe; PD98059, nuclear protein with PD98059 + wild biotin-probe; LY294002, nuclear protein with PD98059 + wild biotin-probe; Scrambled, nuclear protein of transfected miRNA negative control + wild biotin-probe; LNA-182, nuclear protein of transfected LNA-miR-182 inhibitors + wild biotin-probe; LNA-381, nuclear protein of transfected LNA-miR-381 inhibitors + wild biotin-probe; 182M, nuclear protein of transfected miR-182 mimics + wild biotin-probe; 381M, nuclear protein of transfected miR-381 mimics + wild biotin-probe. (G) siRNA-AP2, siRNA-SP1, siRNA-E2F6, and c-Myc overexpression affected endogenous expression of *BRD7* at the protein (left) and mRNA (right) levels. * *p*<0.05 compared with control (LNA-182 and LNA-381 *vs.* scrambled; si-SP1, si-AP2 and si-E2F6 *vs*. si-SC; pCMV-HA-c-Myc *vs*. pCMV-HA).

Previous studies have demonstrated that *LRRC4*, a regulator of miR-182 and miR-381, can inhibit glioma tumorigenicity by modulating receptor tyrosine kinase (RTK) signaling pathways, such as the K-Ras/p-c-Raf/ERK/MAPK and PI-3K/AKT signaling pathways[4], [6]. Therefore, we investigated the signal transduction pathways involved in *BRD7* expression that was inhibited by miR-182 and -381 silencing. Specifically, we analyzed the expression and/or the activation of some of the proteins involved in the K-Ras/p-c-Raf/ERK/MAPK and PI-3K/AKT signaling pathways in response to miR-182 and miR-381 silencing. The Western blot results showed that transfection of LNA-anti-miR-182 and -381, but not of the LNA-scrambled controls, inhibited the expression of K-Ras, p-c-Raf, pERK, PI-3K, and pAKT, but had no effects on N-Ras, total ERK, and AKT expressions (Figure 6A).

Previous studies have also identified some critical transcription factor binding sites in the *BRD7* promoter, namely those for AP2, c-Myc, E2F6, E2F, and Sp1, and confirmed that *BRD7* transcription is positively regulated by SP1 and negatively regulated by c-Myc. We investigated whether miR-182 and miR-381 silencing could induce these transcription factors. First, we examined the effects of different kinase inhibitors (PD98059 for MEK, and LY294002 for PI-3K) on the miR-182- and miR- 381-induced expression of the transcription factors. Our results indicated that, compared with the LNA-anti-scrambled control, transfection of LNA-anti-miR-182 and -381 increased and decreased the expression levels of c-Myc and AP2, SP1, and E2F6 in U251 cells, respectively (Figure 6B). Treatment with PD98059 or LY294002 abrogated the effects induced by the miR-182 and miR-381 mimics (Figure 6C). In addition, to determine whether the miR-182 and miR-381 silencing-induced effects on the expressions of AP2, SP1, E2F6, and c-Myc were *LRRC4*-dependent, we performed *LRRC4* overexpression studies. We found that ectopic overexpression of *LRRC4* in U251 cells (Figure S5A), and endogenous expression of *LRRC4* induced by 5-Aza-dC in U251, SF126, and SF767 cells (Supplementary Figure S5B), resulted in decreased expression of K-Ras, p-c-Raf, pERK, PI-3K, and pAKT, and reversed the expression of AP2, SP1, E2F6, and c-Myc transcription factors. These results suggested that miR-182 and miR-381 silencing modulated the ERK/MAPK and PI-3K/AKT signaling pathways, thereby inhibiting the expression of AP2, SP1, and E2F6 and promoting c-Myc expression.

Next, we investigated the effects of miR-182 and miR-381 silencing on the ability of AP2, SP1, E2F6, and c-Myc to bind to the promoter of *BRD7*. As evidenced by EMSA, transfections of LNA-anti-miR-182 or -381 disrupted the association of AP2, SP1, and E2F6 with the *BRD7* promoter, and promoted association of c-Myc with the *BRD7* promoter (Figure 6D). These transfections also inhibited the activity of the *BRD7* promoter (Figure 6E) and decreased the level of endogenous *BRD7* mRNA and protein (Figure 5B, C). Ectopic overexpression of *LRRC4* in U251 cells (Figure 5D) or endogenous re-expression of *LRRC4* induced by 5-Aza-dC treatment in U251, SF126, and SF767 cells also promoted c-Myc association with the *BRD7* promoter but disrupted the association of AP2, SP1, and E2F6 (Figure S6). Treatment with PD98059 or LY294002 alone, or the two in combination, led to a decrease in AP2, SP1, and E2F6 associating with the *BRD7* promoter and promoted the association of c-Myc with the promoter. Moreover, these kinase inhibitor treatments also reversed the miR-182- and miR-38-induced *BRD7* promoter associations of AP2, SP1, E2F6, and c-Myc (Figure 6F). These results indicated that miR-182 and miR-381 silencing interfered with or promoted the binding of various transcription factors to the *BRD7* promoter. siRNA-mediated silencing of *AP2*, *SP1*, or *E2F6*, and overexpression of *c-Myc* in U251 cells resulted in down-regulation of *BRD7* at both the mRNA and protein levels (Figure 6G).

## Discussion

miRNAs can also function as tumor suppressors or oncogenes to induce cellular transformation and tumorigenesis [23].The facts that miRNAs can regulate the expression of specific genes, including those that are differentially expressed in malignant cells, and that they themselves are differentially expressed in the malignant state, have made miRNAs attractive therapeutic targets [24]. *LRRC4* has been characterized as a tumor suppressor gene involved in glioma formation[4], and our previous study indicated that *LRRC4* is a target of miR-381[17]. In this study, we demonstrated that *LRRC4* is also a target of miR-182. miR-182 and miR-381 were also determined to be involved in the pathological progression of astrocytoma by targeting *LRRC4*. Suppressing endogenous expression of miR-182 and miR-381, respectively, restored the activation of *LRRC4* in gliomas, and inhibited glioma cell proliferation *in vitro* and growth of subcutaneously transplanted tumor *in vivo*. Furthermore, it inhibited the growth of intracranial transplanted tumors, presumably by surpassing the blood-brain barrier. Thus, miR-182 and miR-381 may represent useful therapeutic targets for treatment of these tumors.

The precise roles of miR-182 and miR-381 in relation to LRRC4 expression in gliomas were investigated by the miRNA silencing tool of locked nucleic acids. LNAs are a class of bicyclic conformational analogs of RNA, which exhibit high binding affinity to their complementary RNA molecules and are highly stable *in vivo* [*25*]. In the current study, LNA-mediated miR-182 and miR-381 silencing was applied and found to restore the expression of *LRRC4* in gliomas. We found that LNA-mediated miR-182 and -381 silencing decreased the expression of *BRD7*, which was striking in its similarity to the results obtained with *LRRC4* overexpression. Thus, it was suggested that LNA-mediated miR-182 and miR-381 silencing down-regulated the expression of *BRD7* by restoring *LRRC4* expression in gliomas. BRD7 has been characterized as a potential nuclear transcription factor that regulates cell cycle progression through the Rb/E2F pathways [26], and has been shown to induce cell apoptosis in NPC [27].The study presented herein demonstrated that LNA-mediated miR-182 and miR-381 silencing in gliomas blocked cell cycle progression in the G0/G1 phase by regulating pRb and E2F3 and inhibited cell proliferation *in vitro* and growth *in vivo*. It was also found that LNA-mediated miR-182 and miR-381 silencing induced marked differentiation of tumor cells towards a non-cancerous status.

In previous researches, we had focused on the effect of *LRRC4* on the ERK/MAPK and PI-3K/AKT signaling pathways in gliomas[4], [7], and transcriptional regulation of *BRD7* expression in NPCs [18]. ERK/MAPK and PI-3K/AKT signaling are major cell survival pathways, and play a key role in diverse physiological and pathological processes[25]. In the present study, we demonstrated that LNA-mediated miR-182 and miR-381 silencing can affect the expression and activity of transcription factors that have binding sites in the BRD7 promoter, including AP2, SP1, E2F6, and c-Myc. Moreover, these particular factors are known to be regulated by *LRRC4* through the K-Ras/c-Raf/ERK/MAPK and PI-3K/AKT signaling pathways. miR-182 and miR-381 silencing decreased the expression and activity of AP2, SP1, and E2F6, but increased the expression and activity of c-Myc. Silencing also inhibited the promoter activity and expression of *BRD7*, as did knock-down of AP2, SP1, and E2F6, and overexpression of c-Myc. These results indirectly confirmed that c-Myc is a negative regulator of *BRD7*, and that AP2, SP1, and E2F6 are positive regulators. Of course, this indirect effect may also be explained by a more complex molecular mechanism involving other factors, and we plan to investigate such potential interactions in future studies.

## Materials and Methods

### Cell Culture

Three human glioma-derived cell lines, U251, SF126 and SF767, and the rat C6 glioma cell line were obtained from the Cell Center of Peking Union Medical College in China, and no authentication was performed by the authors since the Cell Center is reputed to be a reliable source with carefully monitored laboratory practices that adhere to international standards. U251 cells were maintained in Dulbecco's Modified Eagle's Medium (DMEM) with 10% fetal calf serum (FCS) and standard antibiotics. SF126, SF767 and C6 cells were cultured in minimum essential medium (MEM). All cells were maintained at 37°C under an atmosphere of 5% CO_2_ and 95% air.

### Patient samples

Sixty-seven human primary brain tumor samples were obtained from randomly selected cancer patients (n = 67) treated at Xiangya Hospital, Hunan, China. All the diagnoses were confirmed by pathology. Written informed consent was obtained from each patient participating in the study prior to surgery. All of the protocols were reviewed and approved by the Joint Ethics Committee of the Central South University Health Authority and performed in accordance with national guidelines.

### Luciferase assays

The 3′-untranslated regions (UTRs) of the *LRRC4* or *BRD7* gene were amplified by PCR from genomic DNA and inserted downstream of the luciferase reporter gene by using the *Hind*III and *Spe*I sites in the pMIR-REPORT miRNA expression reporter vector (Ambion, Shanghai, China). The primer sets used were: 3′-UTR of *LRRC4* containing the miR-182 binding site, 5′-CTAGTCACCATGAATTTGTACTGTGCCAAAATGATAGTGGCAATAATATTTTTCTA-3′ and 5′-AGCTTAGAAAAAT ATTATTGCCACTATCATTTTGGCACAGTACAAATTCATGGTGA-3′; 3′-UTR of *LRRC4* containing the miR-381 binding site, 5′-CTAGTTTGTACAGAGTGGGGAGAGACTTTTTCTTGTATATGCTTATATATTAAGTA-3′ and 5′-AGCTTACTTAATATATAAGCATATACAAGAAAAAGTCTCTCCCCACTCTGTACAAA-3′; and 3′-UTR of *BRD7* containing the miR-381 binding site, 5′-CTAGTGAACAGCGAATTTGGATGTTCCAGAGGTTGGACTTGTATTAGGTAATAAAA -3′ and 5′-AGCTTTTTATTACCTAATACAAGTCCAACCTCTGGAACATCCAAATTCGCTGTTCA-3′. We also generated several inserts with deletions of 4 bp from the site of perfect complementarity of the *LRRC4* and *BRD7*.

The cells were plated into a 24-well plate and incubated for 24 h before transfection. pMIR-REPORT vectors harboring wild-type (WT) or mutant 3′-UTR *LRRC4* sequences were co-transfected into cells along with the miR-182 or miR-381 constructs using Lipofectamine 2000 (Invitrogen, Carlsbad, CA, USA). Luciferase assays were performed 24-48 h after transfection by using the Luciferase Reporter Assay System (Promega, Shanghai, China).

### Real-time quantitative (q)RT-PCR analysis

The total RNAs were extracted from cells or tissues with TRIzol reagent (Invitrogen, Wuhan, China). Reverse transcription reactions were performed with reagents from a SYBR-green-containing PCR kit (GenePharma, Shanghai, China). The primers for qRT-PCR detection of miRNA were designed based on the miRNA sequences provided by the Sanger Center miRNA Registry and were synthesized and purified by Shanghai GenePharma. Human U6 small nuclear (sn)RNA was used for normalization. Primers used are as follows: miR-182, 5′-ACTTTTGGCAATGGTAGAACTCAC-3′ and 5′-AATCCATGAGAGATCCCTAGCG-3′; miR-381, 5′-TAATCTGACTATACAAGGGCAAGCT-3′ and 5′-TATGGTTGT TCTGCTCTCTGTCTC-3′; and U6 snRNA, 5′-ATTGGAACGATACAGAGAAGATT-3′ and 5′-GGAACGCTTCACGAATTTG-3′. The primers for qRT-PCR detection of *LRRC4* or *BRD7* mRNA were synthesized by Invitrogen as follows: *LRRC4*, 5′-GCCGCCATGTTGATTGTC-3′ and 5′-GTGCTGGTTTGTAGGTGTTGTA-3′; and *BRD7*, 5′-TCTTGGGTCCCTCATACA-3′ and 5′-ACTCAGCAACATCCGTCT-3′. mRNA expression was normalized to *β-actin*. Primers for *β-actin* were 5′-AGCGAGCATCCCCCAAAGTT-3′ and 5′-GGGCACGAAGGCTCATCATT-3′. All real-time PCR was performed on the Bio-Rad IQTM5 Multicolor Real-Time PCR Detection System (USA).

### In situ hybridization (ISH), immunochemistry (IHC) and immunofluorescence analysis

miR-182 or miR-381 miRCURY™ LNA custom detection probes (Exiqon, Vedbaek, Denmark) were used for ISH. The 5′-3′ sequence (enhanced with LNA) was TCAGGAACTGCCTTTCTCTCCA or ACAGAGAGCTTGCCCTTGTATA with a DIG label at both the 5′ and 3′ ends. Hybridization, washing, and scanning were carried out according to the manufacturer's instructions. IHC studies were performed using the standard streptavidin/peroxidase staining method as described previously [17]. Image analysis and total gray value estimation were conducted by the GSM-2000P pathology image analysis system (Heima, Zhuhai, China). Immunofluorescence staining was performed as previously described [17]. Stained cells were viewed with a Zeiss LSM510 laser scanning fluorescence microscope.

### miR-182 or miR-381 mimics and LNA-modified Anti-miR-182 or -381 oligonucleotide transfection

The miR-182 mimic (sense: 5′-UUUGGCAAUGGUAGAACUCACACU-3′; anti-sense: 5′-UGUGAGUUCUACCAUUGCUAAAUU-3′), miR-381 mimic (sense: 5′-UAUACAAGGGCAAGCUCUCUGUTT-3′; anti-sense: 5′-ACAGAGAGCUUGCCCUUGUCGCTT-3′), scrambled mimic (sense: 5′-UUCUCCGAACGUGUCACGUTT-3′; anti-sense: 5′-ACGUGACACGUUCGGAGAATT-3′), anti-miR-182 LNA oligonucleotide (5′-LNA-AGUGUGAGUUCUACCAUUGCCAAA-3′), miR-381 LNA oligonucleotide (5′-LNA-ACAGAGAGCUUGCCCUUGUAUA-3′), and scrambled LNA oligonucleotide (5′-LNA-CAGUACUUUUGUGUAGUACAA-3′) were synthesized by GenePharma and were transfected into cells using Lipofectamine 2000.

### 5-Aza-2′-deoxycytidine (5-Aza-dC) demethylation treatment

U251, SF126, and SF767 cells were grown for 48 h in the presence of 10 µM 5-Aza-dC (Sigma-Aldrich, St. Louis, MO, USA), as previously described [5]. Fresh 5-Aza-dC was added every 24 h.

### BRD7 promoter construct

The sequence of BRD7 promoter was previously described in[18].

### BRD7, AP2, SP1, and E2F6 silencing by siRNA

The sense sequences of siRNA oligonucleotides targeting the BRD7, AP2, SP1, and E2F6 transcripts, respectively, were as follows: si-BRD7: 5′-UUUGUUACUGCUUUCAGCGCT-3′; si-AP2: 5′-UUGUUAAUAGGGAUGGCGGTT-3′; si-SP1: 5′-CACAAACACTGCCCACCG-3′; and si-E2F6: 5′-AGGAGACUGGGUAACUUCCTT-3′ (Invitrogen). Scrambled siRNA was used as a negative control. Cells were plated in culture dishes or in 96-well plates for 24 h, and transfected with siRNA using Lipofectamine 2000. After 48 h, the cells were harvested for use in other assays or for extractions of RNA and protein.

### Western blot

Western blot analysis was performed as previously described[4]. Primary antibodies against p44/42 MAPK (Erk1/2) (Thr202/Tyr204), pAkt (Ser-473), total ERK, AKT, pRaf, and GAPDH purchased from Cell Signaling Technology (Beverly, MA, USA). The SP1 antibody was purchased from Upstate Biotechnology (Lake Placid, NY, USA). The K-Ras, c-Myc, pRb, and E2F6 antibodies were purchased from Santa Cruz Biotechnology (Santa Cruz, CA, USA). The anti-LRRC4 monoclonal antibody was generated in our lab. The intensity of protein fragments was quantified using Chemical-QDocTM XRSt (Bio-Rad).

### Electrophoretic mobility shift assays (EMSA)

Complementary oligonucleotides derived from the human *BRD7* promoter regions and containing putative Sp1, E2F6, c-Myc and E2F-binding sites, as previously described [19], were synthesized by Invitrogen. Nuclear extracts were isolated from cells using the Nuclear and Cytoplasmic Extraction Reagent (Pierce Chemical, Rockford, IL, USA). EMSAs with nuclear extracts and biotin-labeled *BRD7* promoter oligonucleotides were performed according to the LightShift Chemiluminescent EMSA Kit's instructions (Pierce Chemical).

### Cell proliferation assay

This procedure was carried out as previously described[4].

### Cell cycle assay

This procedure was carried out as previously described [4].

### Subcutaneously transplanted tumor formation assay in nude mice

This procedure was carried out as previously described [17].

### Intracranial transplanted tumor model in rats

All experiments were carried out with the approval of the Animal Care and Use Committee of Central South University. Sprague-Dawley rats, 4–6 weeks old and weighing 200–250 g were used. For implantation to brain parenchyma, C6 glioma cells were suspended at a concentration of 2×10^6^ cells in 10 µL PBS. Rats were anesthetized by intraperitoneal injection of ketamine (40 mg/kg) in combination with xylazine (10 mg/kg). Each rat's head was mounted in a stereotactic head holder and a burr hole was made 4 mm from the brain surface, 4 mm lateral and 0.5 mm posterior to the Bregma for determining cerebral blood flow in the subcortex region. A stainless cannula was inserted through the hole into the S1 region to make an injection pocket. Cells were injected via a stereotactic pump at 1 µL/min using a 25 µL syringe. After injection of the cells, the needle was allowed to remain inside the tissue for 5 min to prevent the cell suspension from leaking out. After the syringe was extracted, the hole was sealed with sterile bone wax. Strict sterility was maintained throughout the procedure.

### In vivo magnetic resonance imaging

On the 7th day after implantation of C6 glioma cells, rats were randomly divided into four groups (n = 5 per group). Aliquots of 200 µL of 0.9% saline solution containing 2 µg of scrambled, LNA-anti-miR-182, or LNA-anti-miR-381, or 1 µg of LNA-anti-miR-182+1 µg of LNA-anti-miR-381 were administered via intraperitoneal injection for one day. On the 28th day after implantation of the C6 glioma cells, rats were anesthetized as described above and intravenously injected with 0.2 mL/kg gadopentetic acid dimeglumine salt injection (Bayer Schering Pharma, Germany). MRI was carried out for T1WI with FSE sequence and T2WI with FSE sequence, with section thickness of 2 mm and gap of 0.1 mm. Tumor volume was calculated as follows: V = L×l^2^×0.5 mm^3^, where L and l represented the larger and smaller tumor diameters, respectively.

### Statistical analysis

Differences between groups were tested by the Student's *t*-test or one-way ANOVA using the SPSS 11.0 program (Chicago, IL, USA). Spearman's correlation test was used to evaluate the pairwise expression correlation between miR-182, miR-381, *BRD7* and *LRRC4* in gliomas. A *p*-value of less than 0.05 was considered statistically significant.

## Supporting Information

Figure S1 **miR-381 did not combine with the 3′-UTR of BRD7.**
(TIF)

Figure S2 **qRT-PCR analysis showing miRNA-182 and miR-381 expression in normal brain and WHO grade I, II, III astrocytomas, and grade IV glioblastoma.**
(TIF)

Figure S3 **Compared to the LNA-mediated miR-182 and -381 silencing or treatment with the DNA demethylating agent 5-Aza-dC, combination of miR-182 and miR-381 silencing, or miR-182 and miR-381 silencing and 5-Aza-dC did not promote the proliferation of glioma cells.**
(TIF)

Figure S4 (A) Intraperitoneal injection of LNA-anti-miR-182 and/or -381 oligonucleotides surpassed the blood-brain barrier in Sprague-Dawley rats and inhibited the growth of intracranial transplanted tumors. *p<0.05 vs. control (LNA-scramble); #p<0.05 vs. LNA-anti-miR-182 or LNA-anti-miR-381. (TIF) (B) Quantitation of the total gray value of miR-182, miR-381, LRRC4, BRD7 and Ki-67. Image analysis and total gray value were estimated by the GSM-2000P pathology image analysis system.
(TIF)

Figure S5 (A) Western blot showing the effect of LRRC4 overexpression in U251 cells on expressions of signaling factors K-Ras, p-c-Raf, pERK, PI-3K and transcription factors pAKT, AP2, SP1, E2F6 and c-Myc. (TIF) (B) Western blot showing the effect of endogenous LRRC4 expression induced by 5′-Aza-dC in U251, SF126 and SF767 cells on expressions of signaling factors K-Ras, p-c-Raf, pERK, PI-3K and transcription factors pAKT, AP2, SP1, E2F6 and c-Myc.
(TIF)

Figure S6 **EMSA confirming that endogenous expression of LRRC4 induced by 5′-Aza-dC in U251, SF126 and SF767 cells disrupts the association of AP2, SP1and E2F6 with the BRD7 promoter and promotes c-Myc association.**
(TIF)
