# Supplementary figures and images for: Disturbing miR-182 and -381 Inhibits BRD7 Transcription and Glioma Growth by Directly Targeting LRRC4
Source: PLoS One. 2014 Jan 3;9(1):e84146. doi: 10.1371/journal.pone.0084146 (PMC3880275; doi:10.1371/journal.pone.0084146)

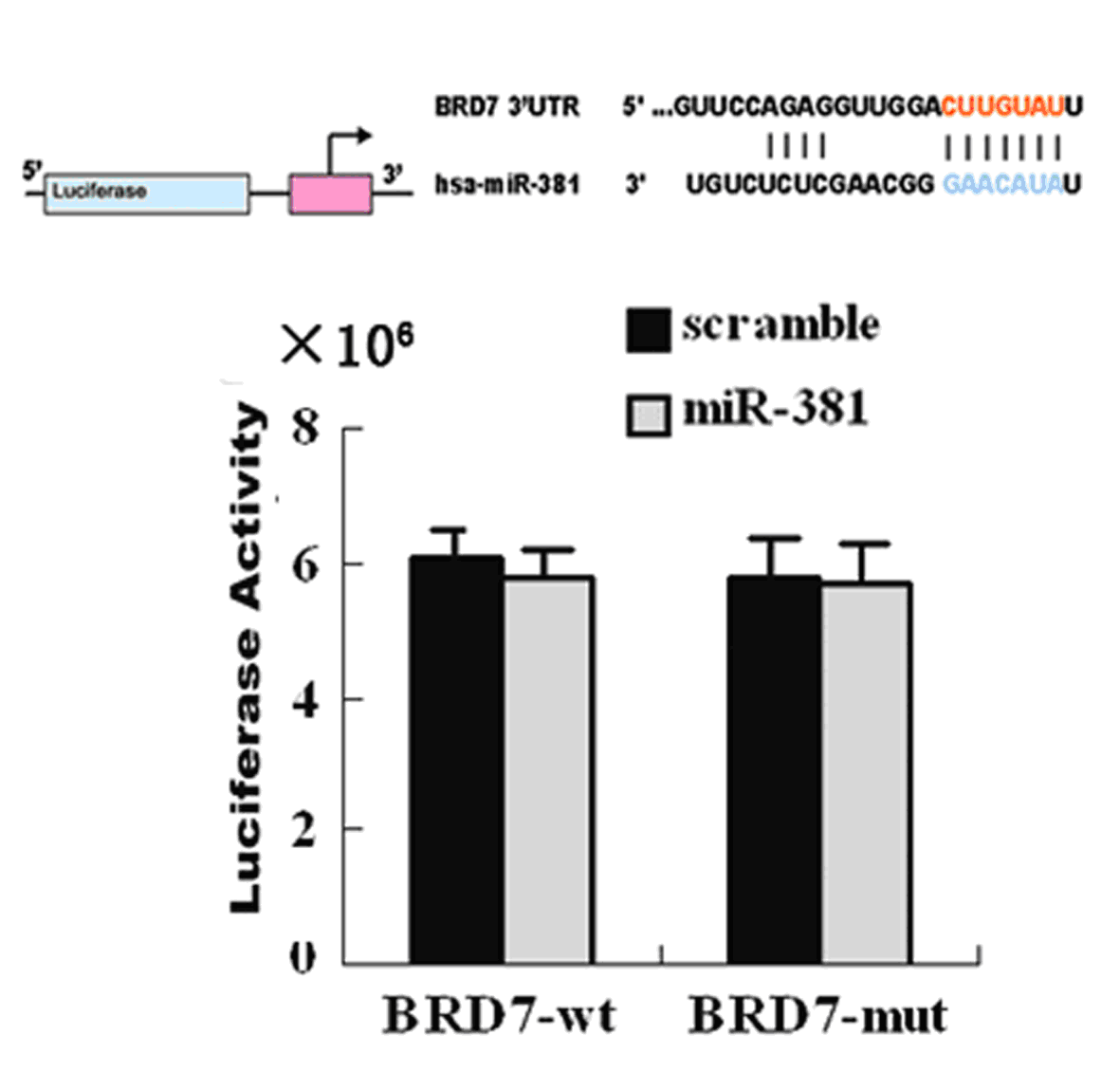

Supplement: Figure S1 — miR-381 did not combine with the 3′-UTR of BRD7. (TIF) [file pone.0084146.s001.tif]

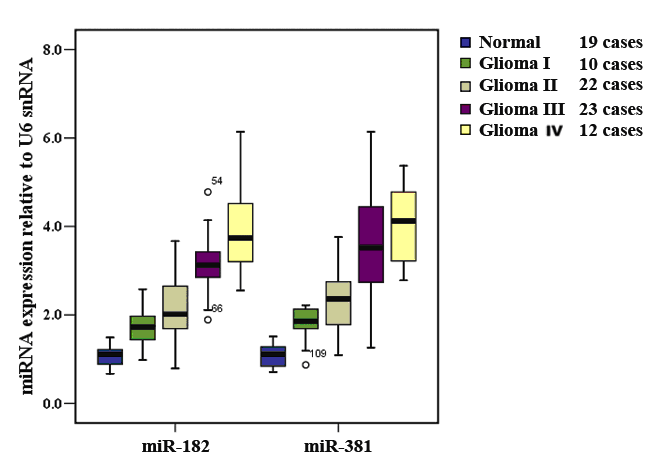

Supplement: Figure S2 — qRT-PCR analysis showing miRNA-182 and miR-381 expression in normal brain and WHO grade I, II, III astrocytomas, and grade IV glioblastoma. (TIF) [file pone.0084146.s002.tif]

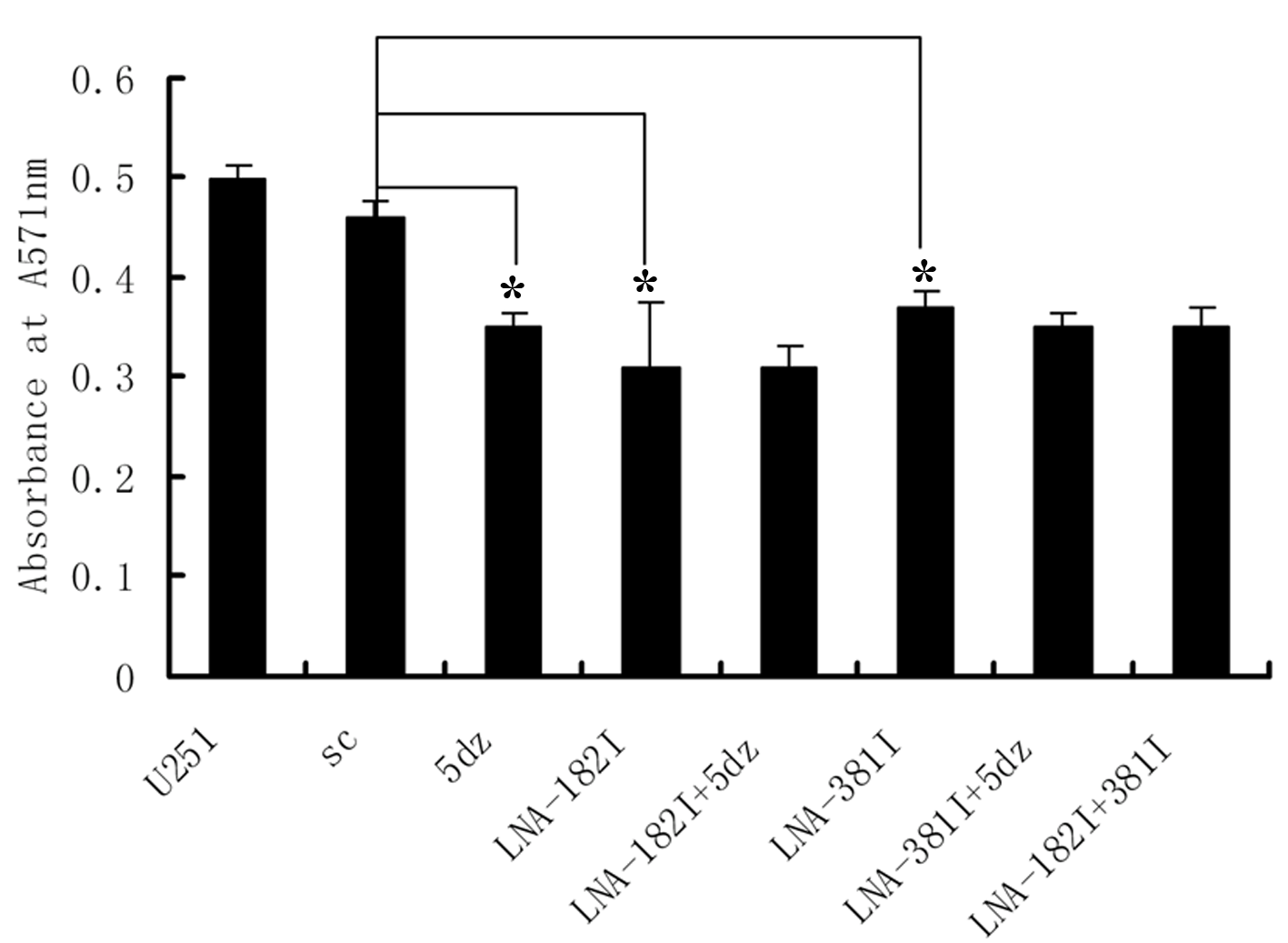

Supplement: Figure S3 — Compared to the LNA-mediated miR-182 and -381 silencing or treatment with the DNA demethylating agent 5-Aza-dC, combination of miR-182 and miR-381 silencing, or miR-182 and miR-381 silencing and 5-Aza-dC did not promote the proliferation of glioma cells. (TIF) [file pone.0084146.s003.tif]

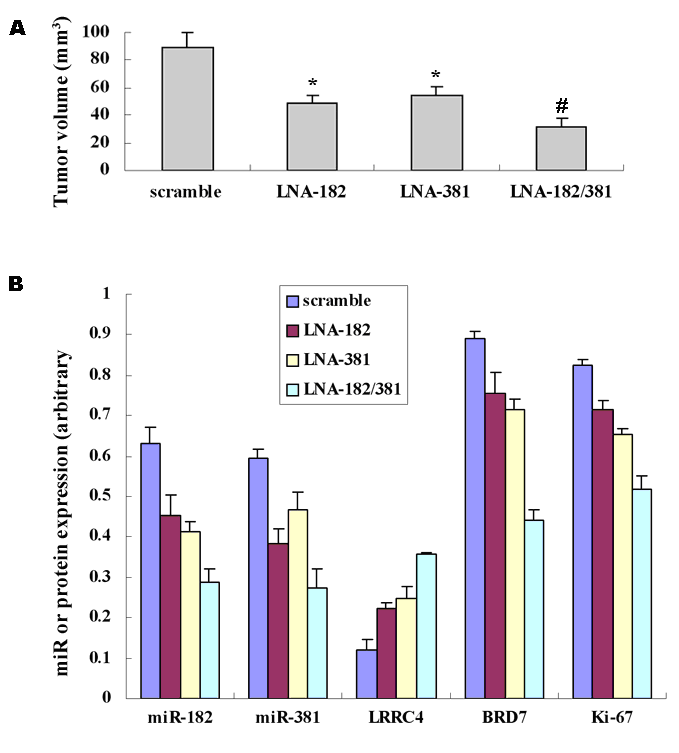

Supplement: Figure S4 — (A) Intraperitoneal injection of LNA-anti-miR-182 and/or -381 oligonucleotides surpassed the blood-brain barrier in Sprague-Dawley rats and inhibited the growth of intracranial transplanted tumors. *p<0.05 vs. control (LNA-scramble); #p<0.05 vs. LNA-anti-miR-182 or LNA-anti-miR-381. (TIF) (B) Quantitation of the total gray value of miR-182, miR-381, LRRC4, BRD7 and Ki-67. Image analysis and total gray value were estimated by the GSM-2000P pathology image analysis system. (TIF) [file pone.0084146.s004.tif]

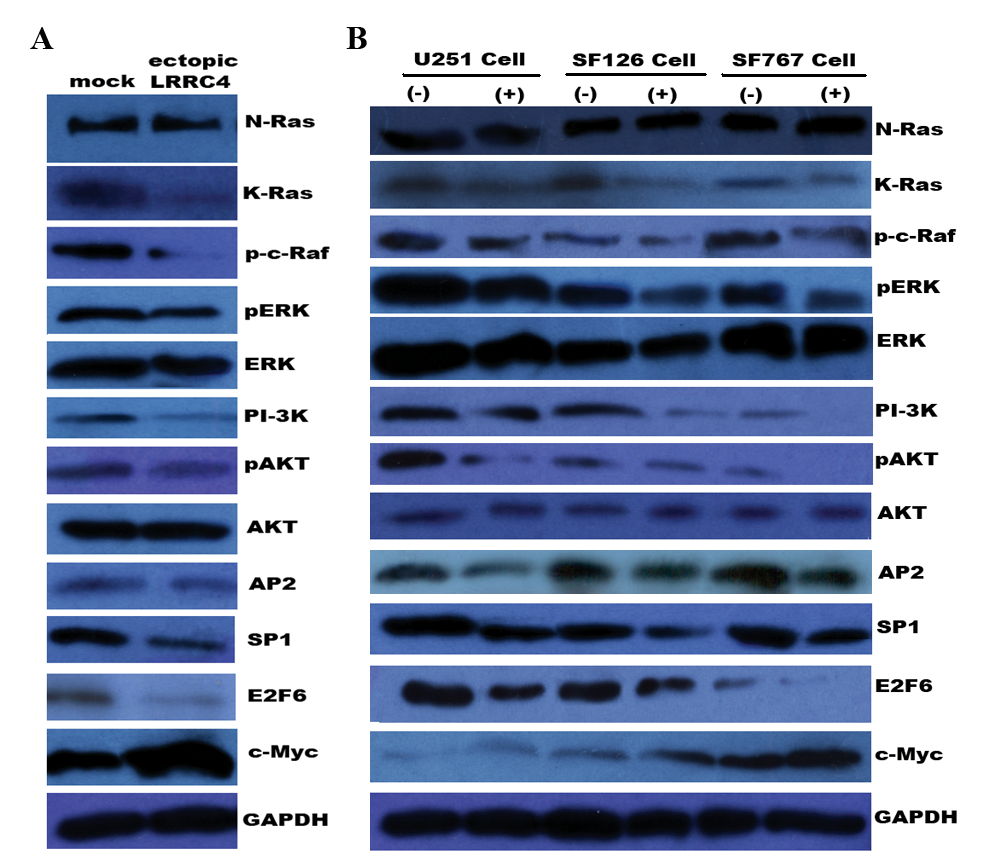

Supplement: Figure S5 — (A) Western blot showing the effect of LRRC4 overexpression in U251 cells on expressions of signaling factors K-Ras, p-c-Raf, pERK, PI-3K and transcription factors pAKT, AP2, SP1, E2F6 and c-Myc. (TIF) (B) Western blot showing the effect of endogenous LRRC4 expression induced by 5′-Aza-dC in U251, SF126 and SF767 cells on expressions of signaling factors K-Ras, p-c-Raf, pERK, PI-3K and transcription factors pAKT, AP2, SP1, E2F6 and c-Myc. (TIF) [file pone.0084146.s005.tif]

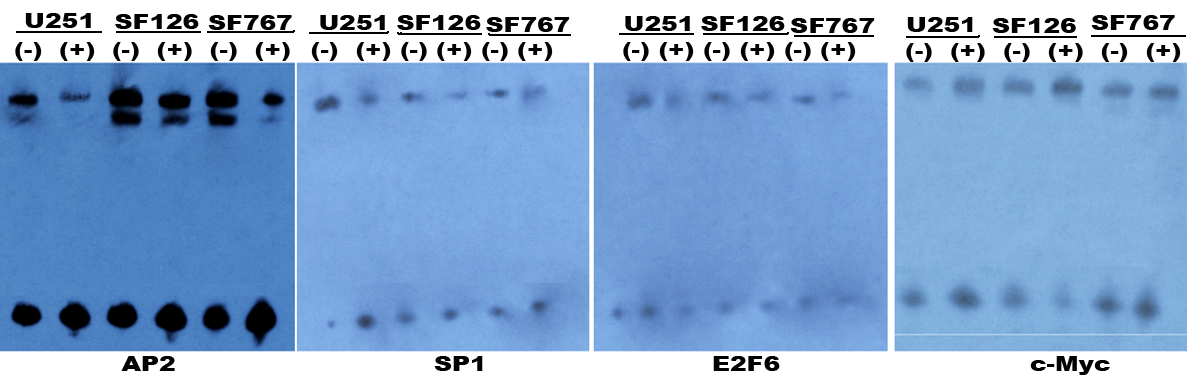

Supplement: Figure S6 — EMSA confirming that endogenous expression of LRRC4 induced by 5′-Aza-dC in U251, SF126 and SF767 cells disrupts the association of AP2, SP1and E2F6 with the BRD7 promoter and promotes c-Myc association. (TIF) [file pone.0084146.s006.tif]
